# Supplementary figures and images for: Noise leads to the perceived increase in evolutionary rates over short time scales
Source: PLoS Comput Biol. 2024 Sep 13;20(9):e1012458. doi: 10.1371/journal.pcbi.1012458 (PMC11424004; doi:10.1371/journal.pcbi.1012458)

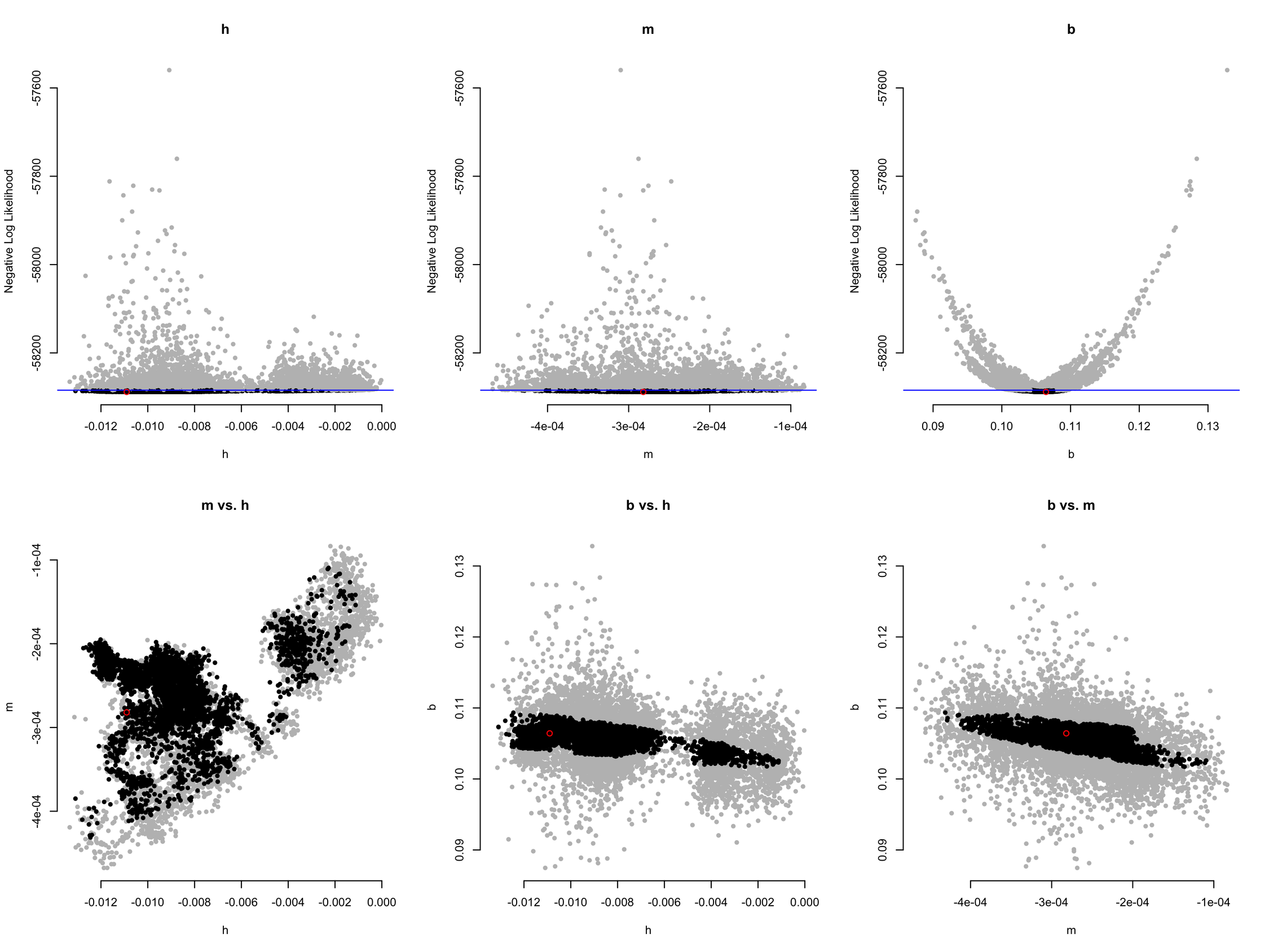

Supplement: S1 Fig — The top row displays univariate confidence regions for each parameter fitted to the Yule simulation dataset. The best model, denoted as hmb, allowed all parameters to vary freely. Sampled parameter values are represented by dots, with gray dots indicating values outside the confidence region and black dots inside. The blue line indicates the boundary between these points. This representation offers a flattened view of the multidimensional analysis; within the horizontal range of black points, some gray points may exist where values for other parameters lead to poor likelihoods. The red circle marks the maximum likelihood estimate. The second row presents bivariate plots, with colors indicating confidence regions as described above. In an effective analysis, the black region in these plots should form an ellipsoid shape, surrounded by gray. To obtain a conservative estimate from the points, such as those in the rightmost column of Fig 1 in the main text, a rectangular prism was placed around the clusters of black points. The range of values, such as the proportion of hyperbolic weight, was derived from calculations at the vertices of this prism. The overall approach of dentist resembles Markov Chain Monte Carlo in Bayesian analysis but does not rely on prior distributions. Instead, it focuses on establishing bounds rather than defining a region that comprises a certain proportion of overall probability. (TIF) [file pcbi.1012458.s001.tif]

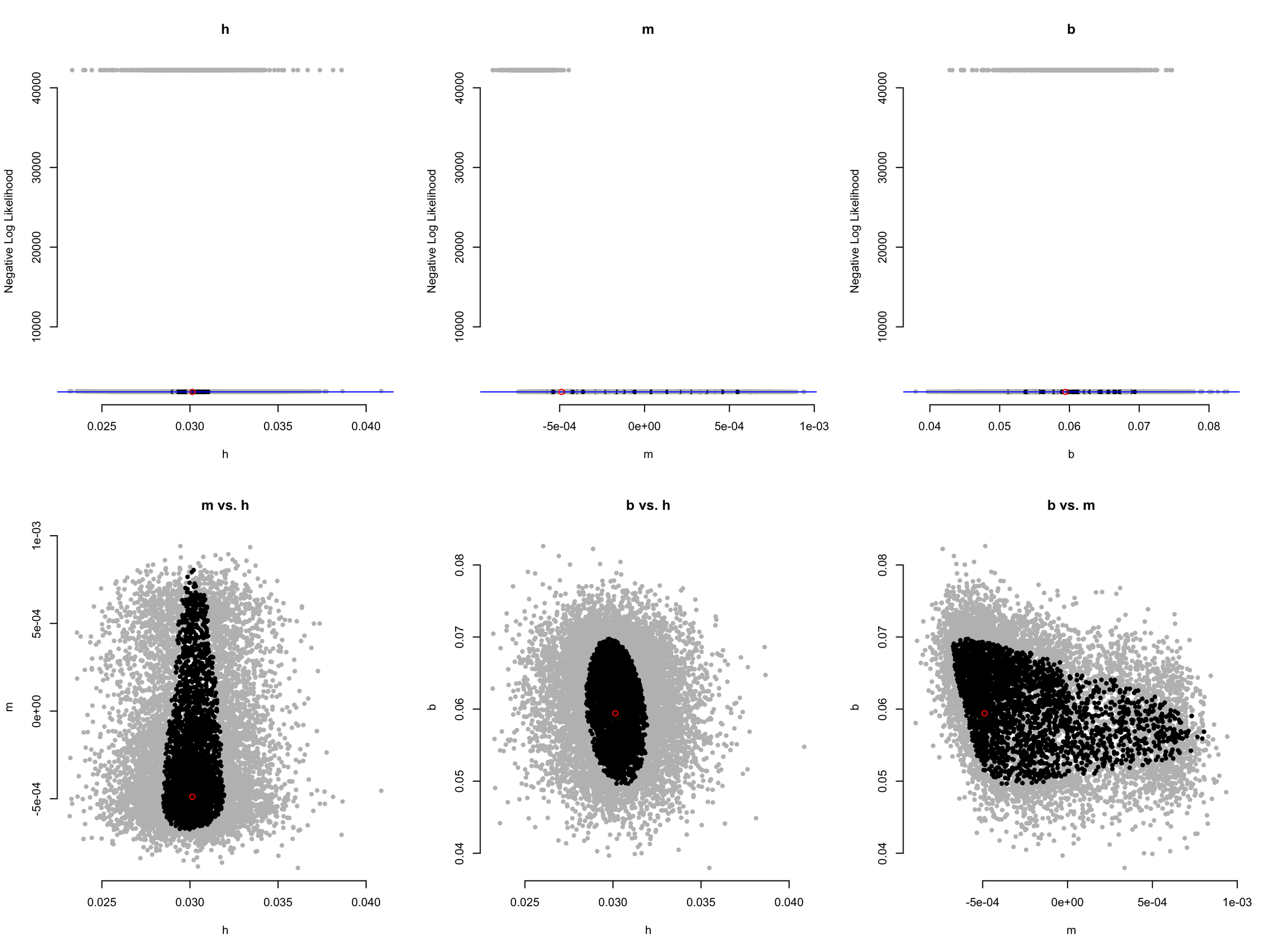

Supplement: S2 Fig — The best model was hmb so all parameters were free to vary. (TIF) [file pcbi.1012458.s002.tif]

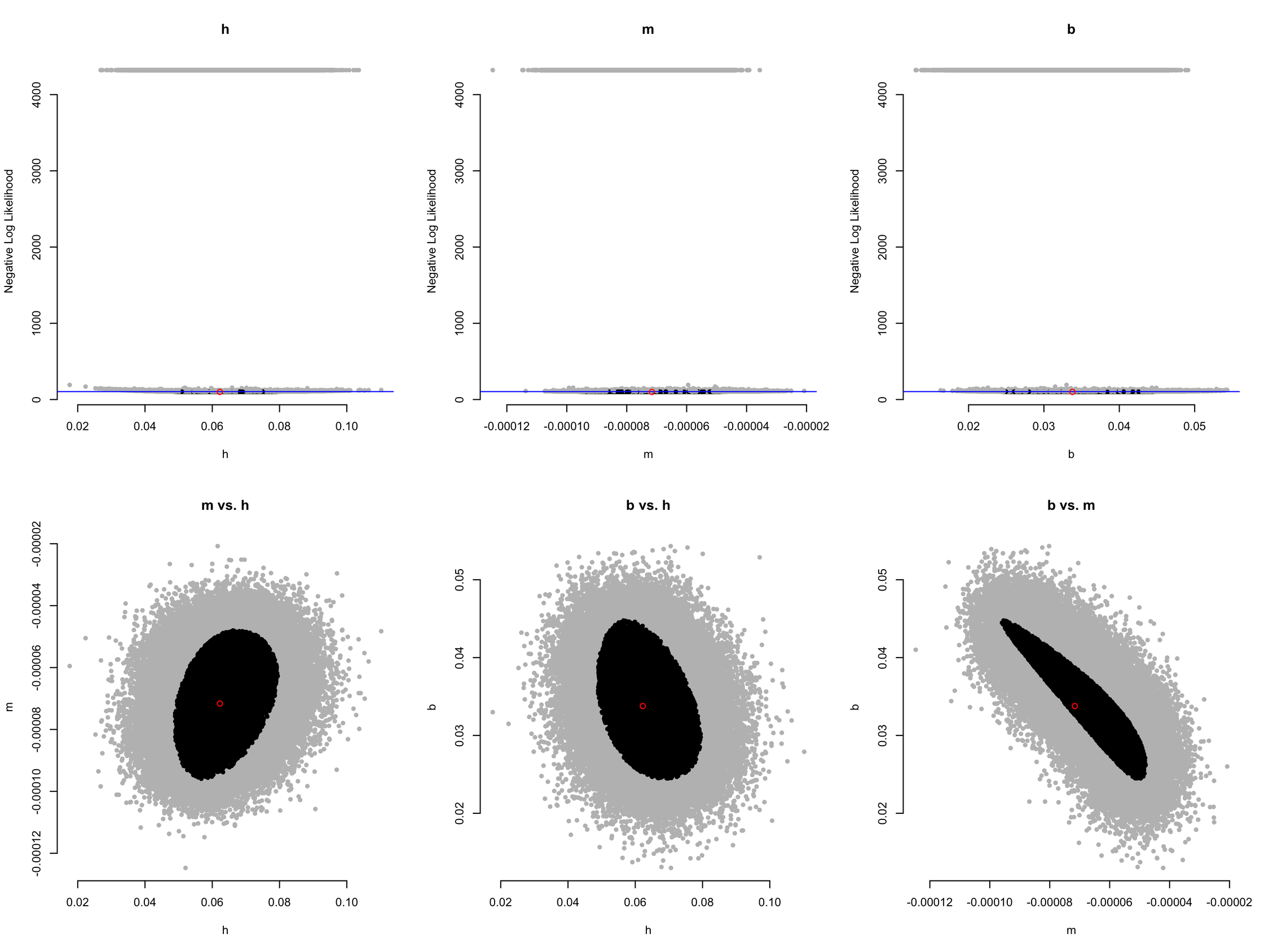

Supplement: S3 Fig — The best model was hmb so all parameters were free to vary. (TIF) [file pcbi.1012458.s003.tif]

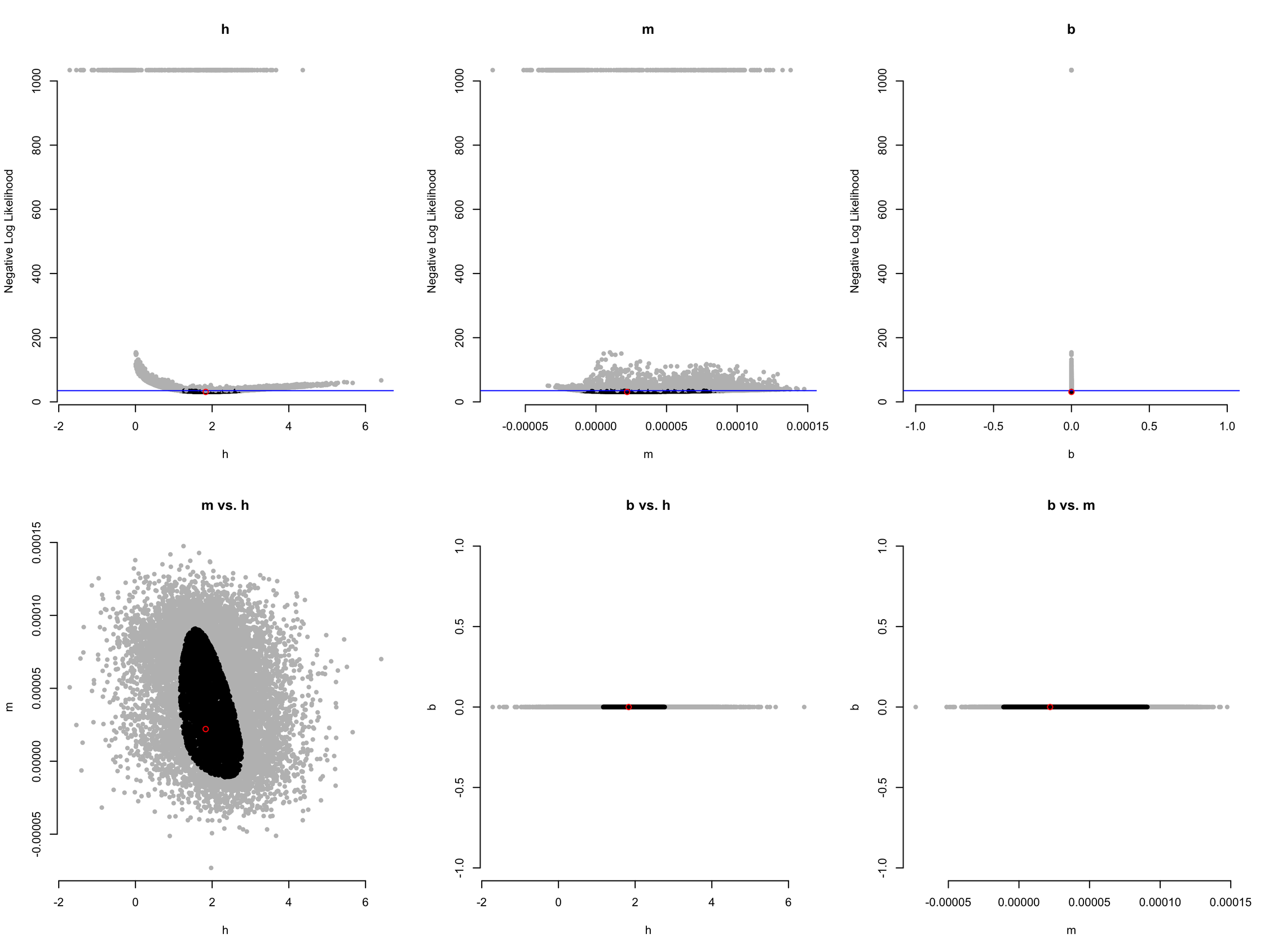

Supplement: S4 Fig — The best model was hm0 so the b parameter is fixed at zero. (TIF) [file pcbi.1012458.s004.tif]

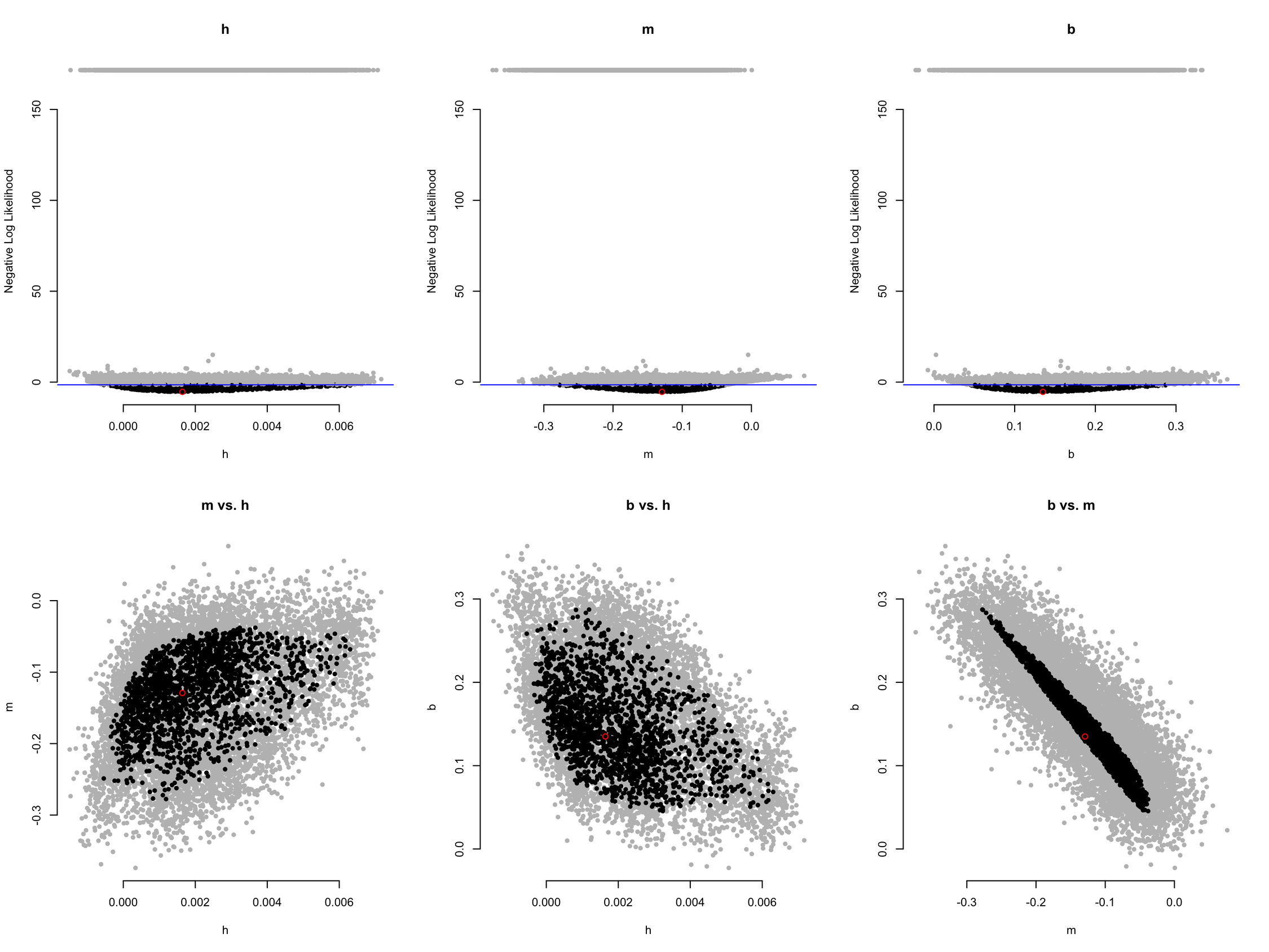

Supplement: S5 Fig — The best model was hmb so all parameters were free to vary. (TIF) [file pcbi.1012458.s005.tif]

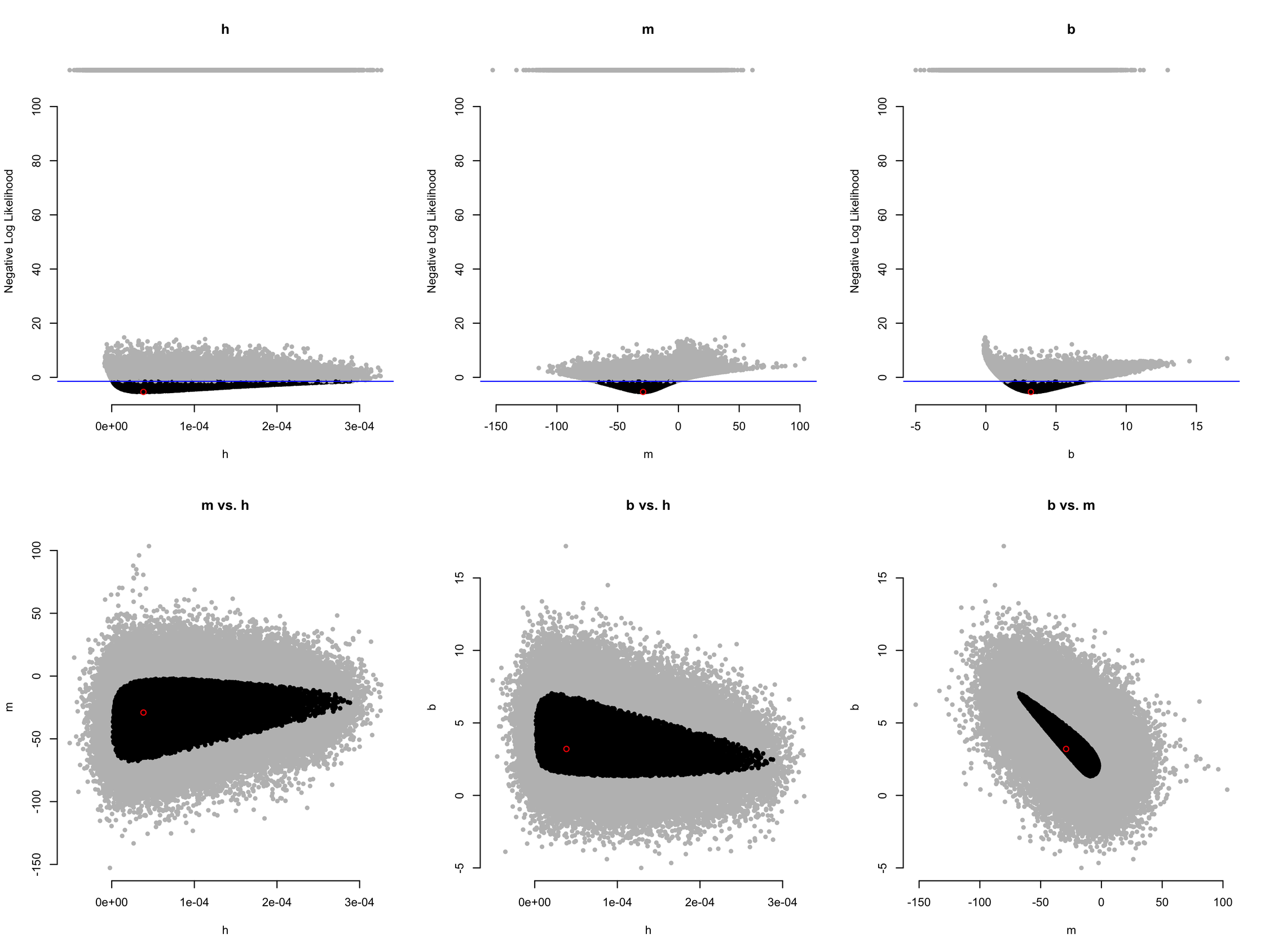

Supplement: S6 Fig — The best model was hmb so all parameters were free to vary. (TIF) [file pcbi.1012458.s006.tif]

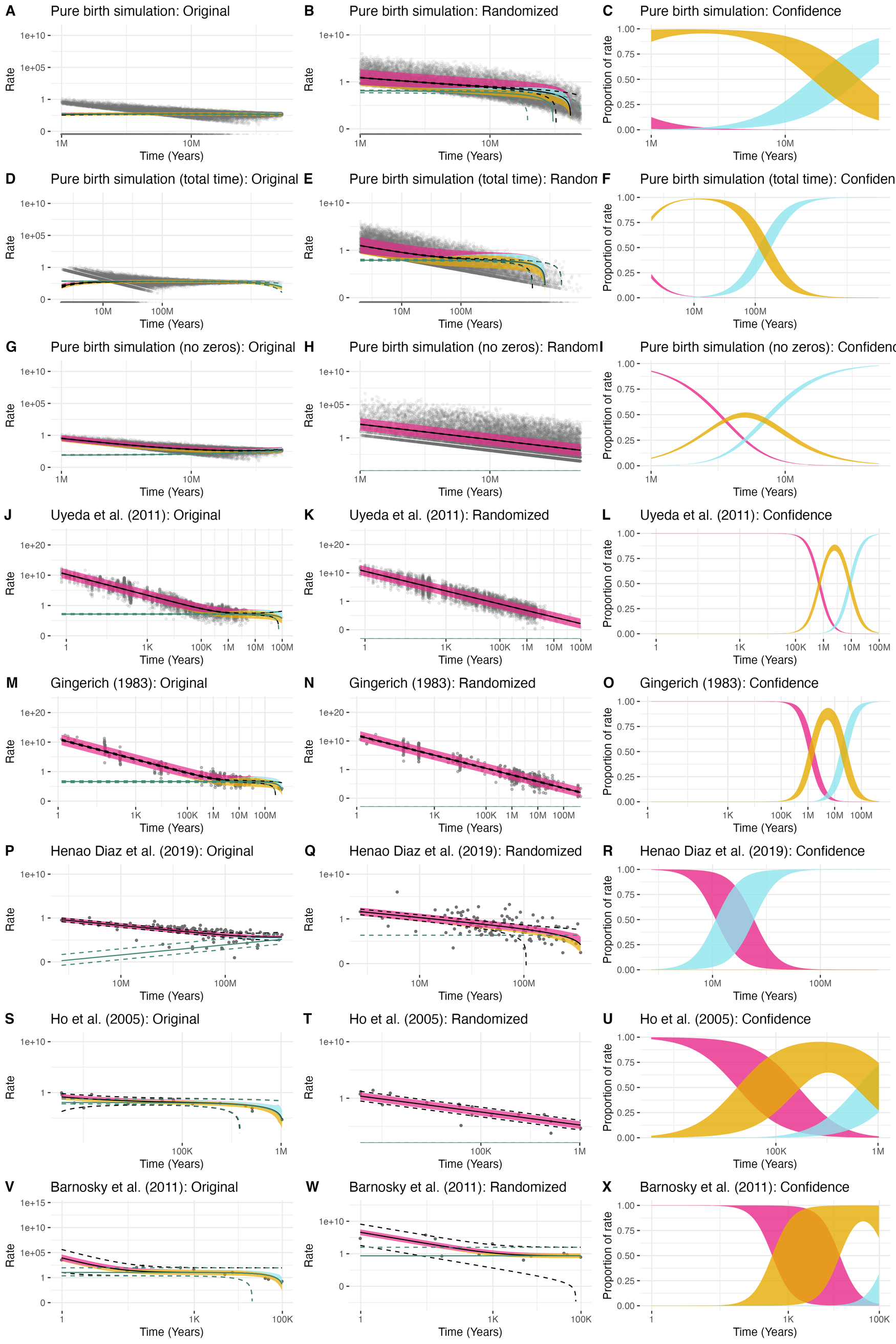

Supplement: S7 Fig — The first column is for the original data; the second column is using the randomization procedure. The horizontal axis is time; the vertical axis is rate (both on a log-scale). Dots show individual rate estimates; the black line shows the regression from the best fitting model and the dashed lines show the 95% confidence interval around that regression. The thick line shows the relative impact of the magnitude of each component on the overall rate in the best fitting model: dark red is from the hyperbolic component (which would be linear on this log-log plot), goldenrod is from a constant rate component, and blue is from a linear component. Note that for the pure birth simulation there are some rates that are zero, which are placed at negative infinity on a log scale; ggplot2 handles these by plotting them along the x-axis. The third column shows the proportion of the rate from each component over time, with the thickness of the bands representing the uncertainty in that proportion. (TIF) [file pcbi.1012458.s007.tif]

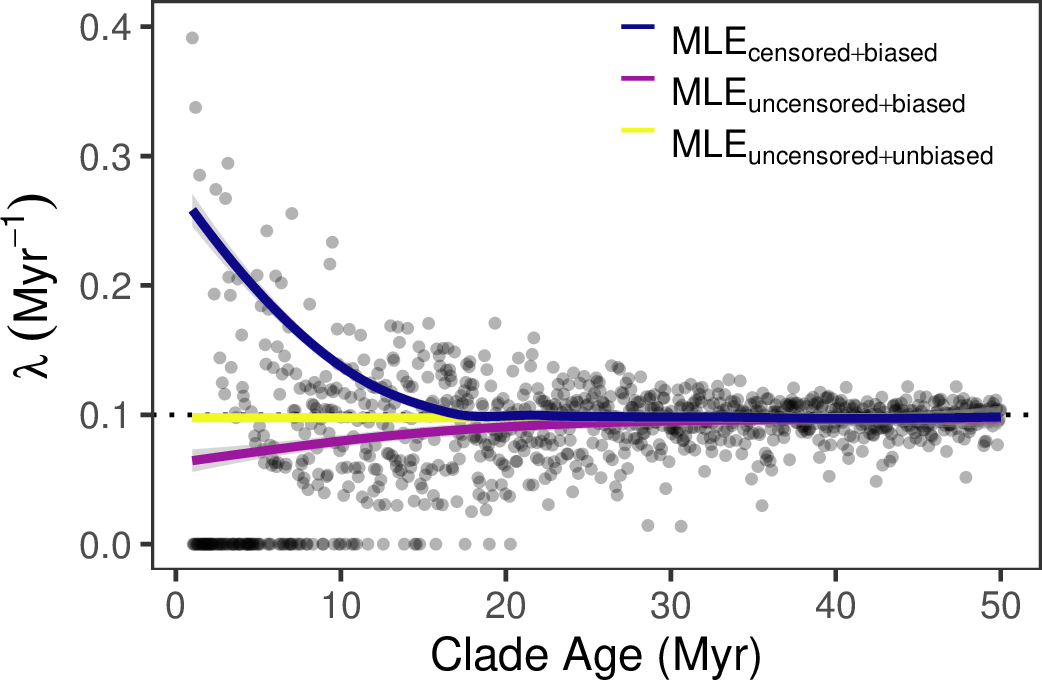

Supplement: S8 Fig — We simulated 1000 Yule trees that assumed a constant rate of 0.1 birth My-1, (dotted black line) regardless of the time. When zero rate tree are excluded (i.e., censored), and we rely on the MLE of the birth rate, λ^, we find that rates exponentially increase towards the present (blue line, MLEcensored+biased). However, when we include the zero rate trees the line dramatically shifts downwards, with rates nearer the present being lower than in deeper time frames (purple line, MLEuncensored+biased). This suggests that the MLE estimator of the birth is biased. When we apply a “correction” to account for the bias, we see that the rates are indeed constant through time (yellow line, MLEuncensored+unbiased), which is consistent with the simulation scenario. (TIF) [file pcbi.1012458.s008.tif]

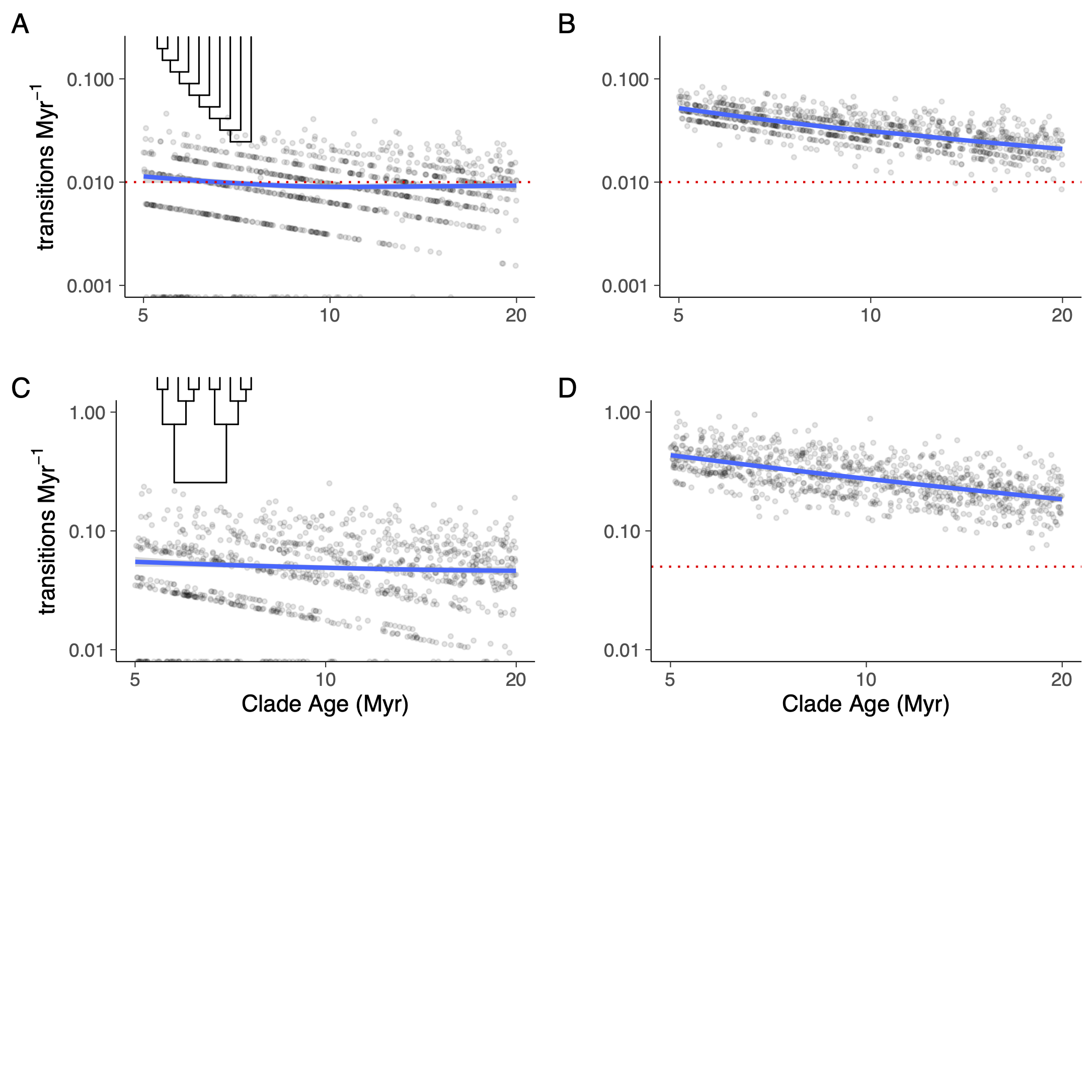

Supplement: S9 Fig — The figure displays the results of state transition rates estimated at various time points, using data simulated on two types of 64-taxon trees representing extremes in tree balance: pectinate (resembling “caterpillar” trees) and perfectly balanced trees, where diversity is evenly distributed between sister clades. The true rates were assumed to be constant over time, indicated by the red dashed line. The first column (a and c) illustrates rates with no errors in the datasets and constant rates over time, depicted by the blue trend line. The second column (b and d) shows rate estimates from the same data but with errors introduced by randomly selecting several taxa and changing their true state to the incorrect one before re-estimating the rate. These errors result in an artificial negative relationship between the transition rate and clade age. (TIF) [file pcbi.1012458.s009.tif]

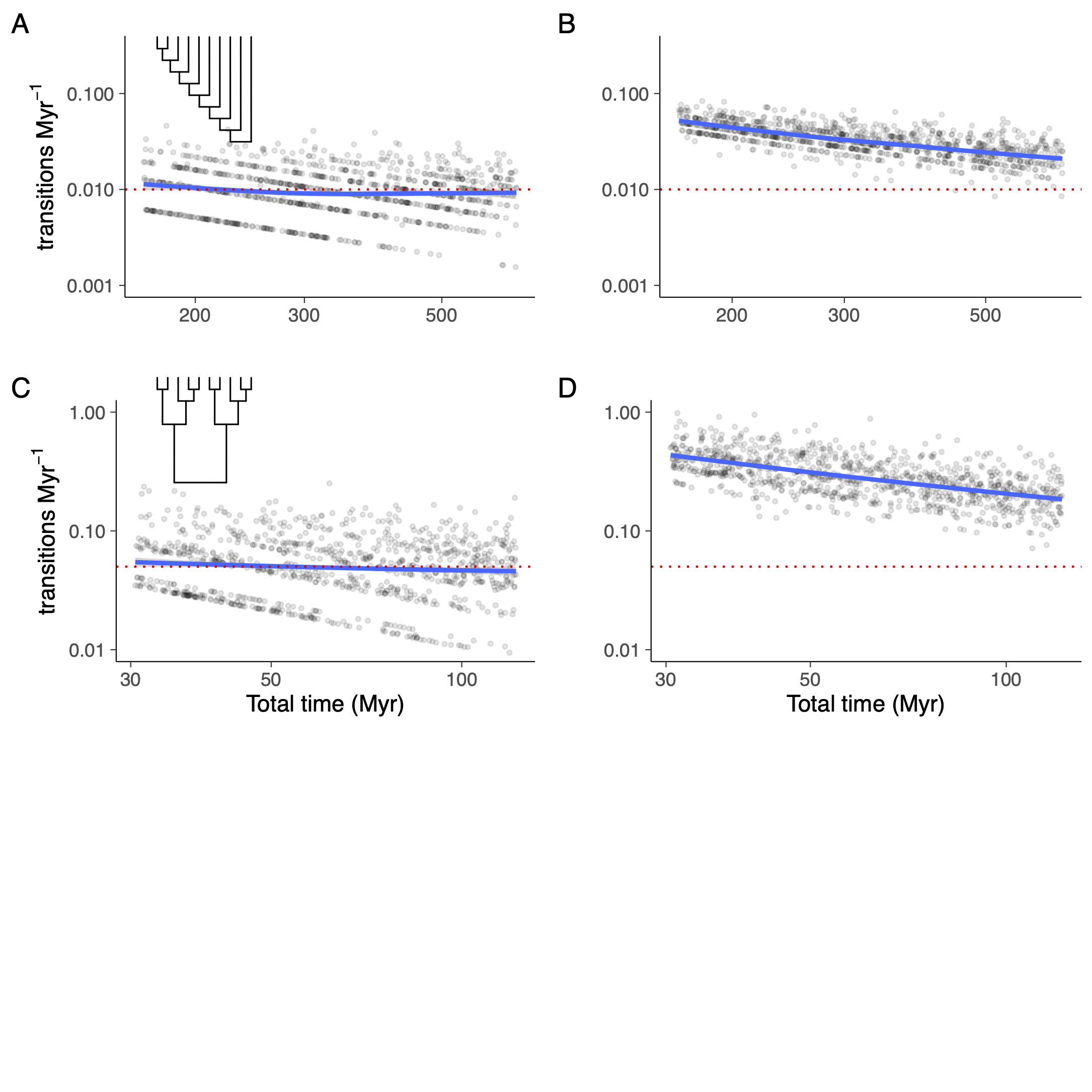

Supplement: S10 Fig — Note that this figure is similar to S9 Fig but replaces clade age on the vertical axis with the total time represented by a given tree (i.e., the sum of all branch lengths). Note the difference in scale for both axes. Pectinate trees are less affected by errors compared to balanced trees due to the greater overall time represented in the former. (TIF) [file pcbi.1012458.s010.tif]

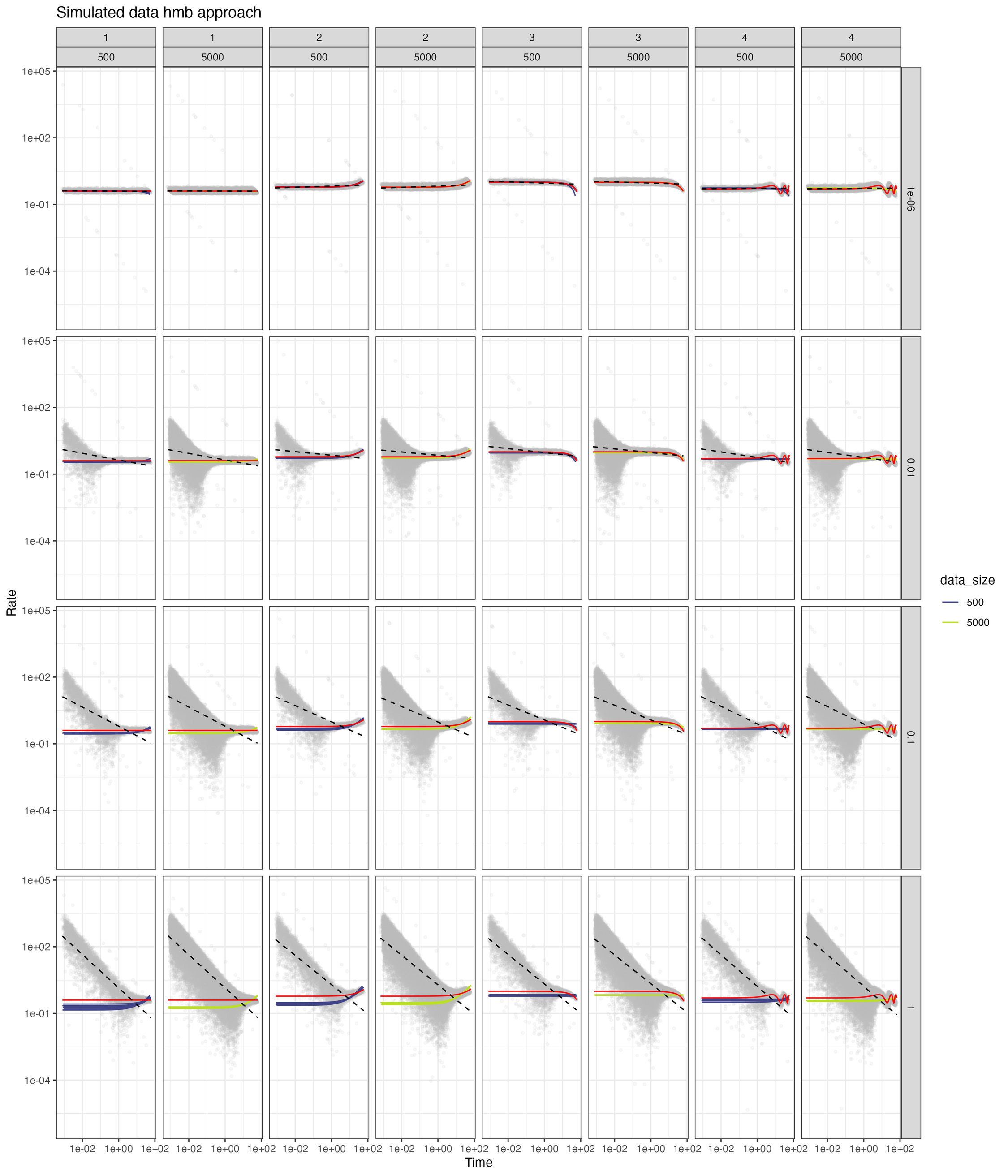

Supplement: S11 Fig — Each dot represents a simulated comparison. Within each subplot, the x-axis represents time and the y axis empirical rate, both on a log scale. The red line represents the true rate at that time under the model, the other solid lines the reconstructed rates under the best-fitting hmb model for each replicate dataset. The dashed line shows a linear fit to the points. The subgraphs are arranged in columns by generating models 1, 2, 3, and 4 and by dataset sizes of 500 or 5000 points and in rows by the standard deviation used in the simulation. Model 1 is a constant rate; models 2 and 3 are increasing and decreasing rates with increased time, respectively; model 4 is a sine wave with a periodicity of 26 million years. Note that with low measurement error, the clouds of points resemble the generating model (red line) but with increasing amounts of noise the clouds begin to resemble the points found in the empirical datasets. (TIF) [file pcbi.1012458.s011.tif]

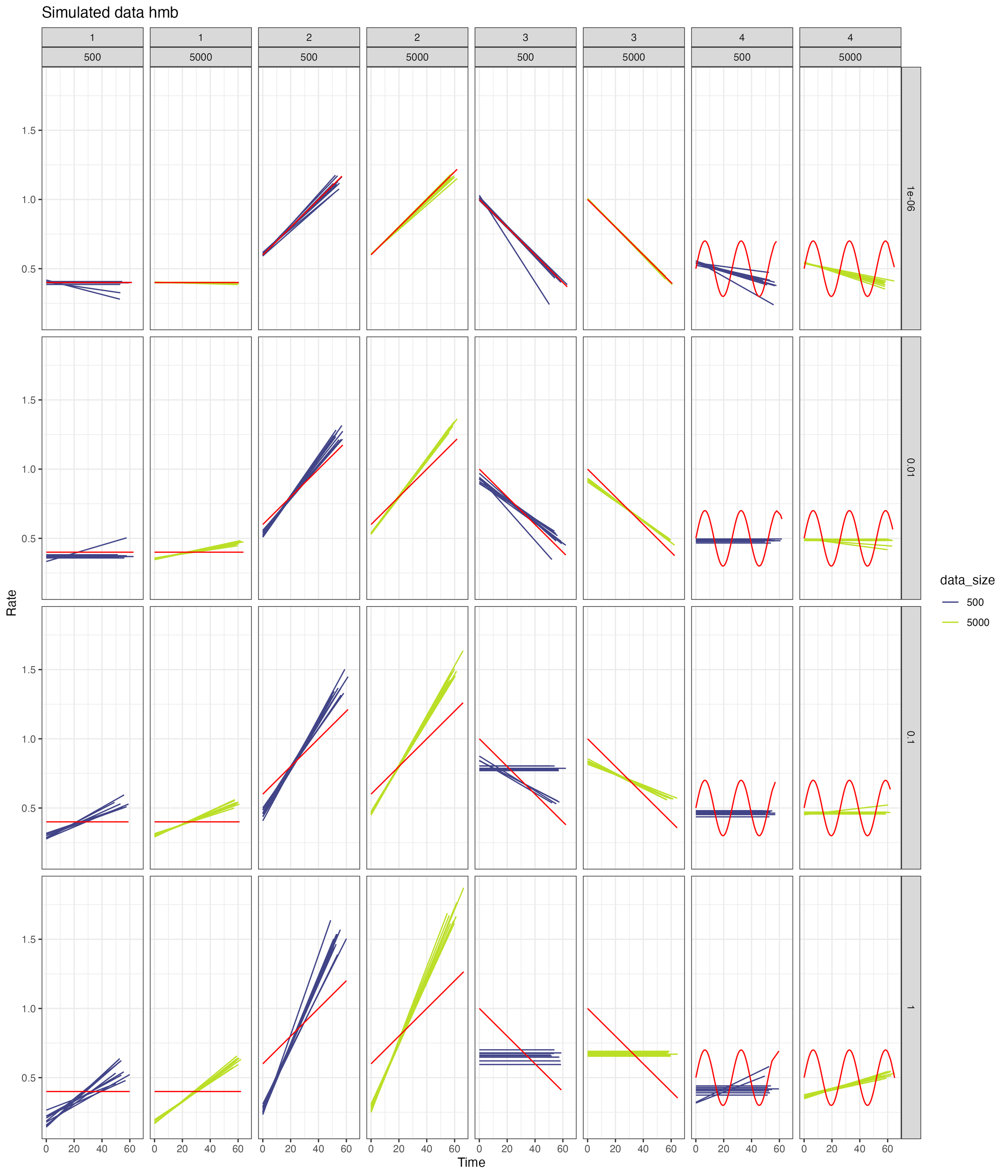

Supplement: S12 Fig — Each purple or green line represents the hmb model fit for the generating rate from a different replicate; the true generating rate is in red. Note that the axes in this plot are not log transformed. (TIF) [file pcbi.1012458.s012.tif]

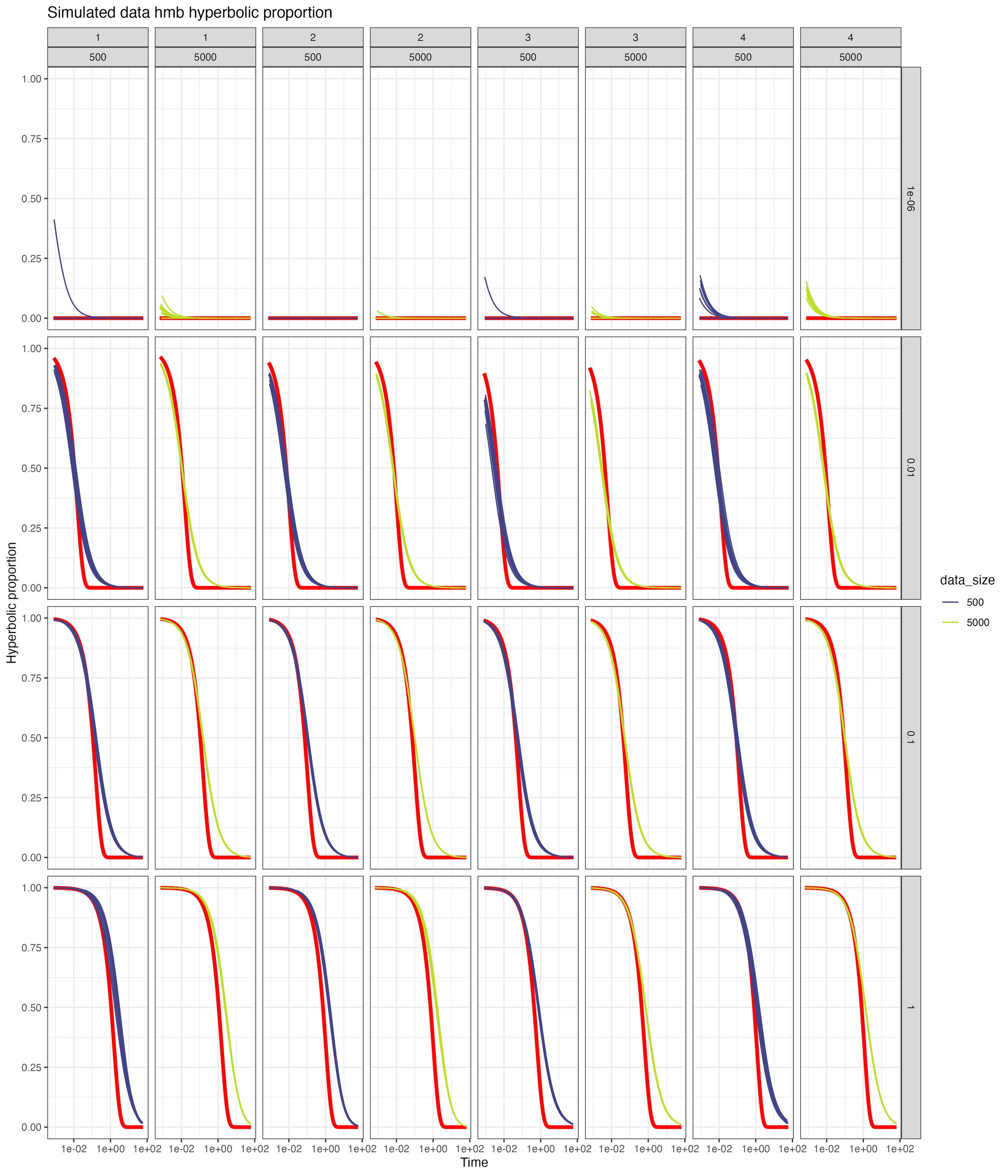

Supplement: S13 Fig — The match is not perfect, but hmb generally performs well despite in some cases (like model 4) not being able to match the complexity of the generating model. (TIF) [file pcbi.1012458.s013.tif]

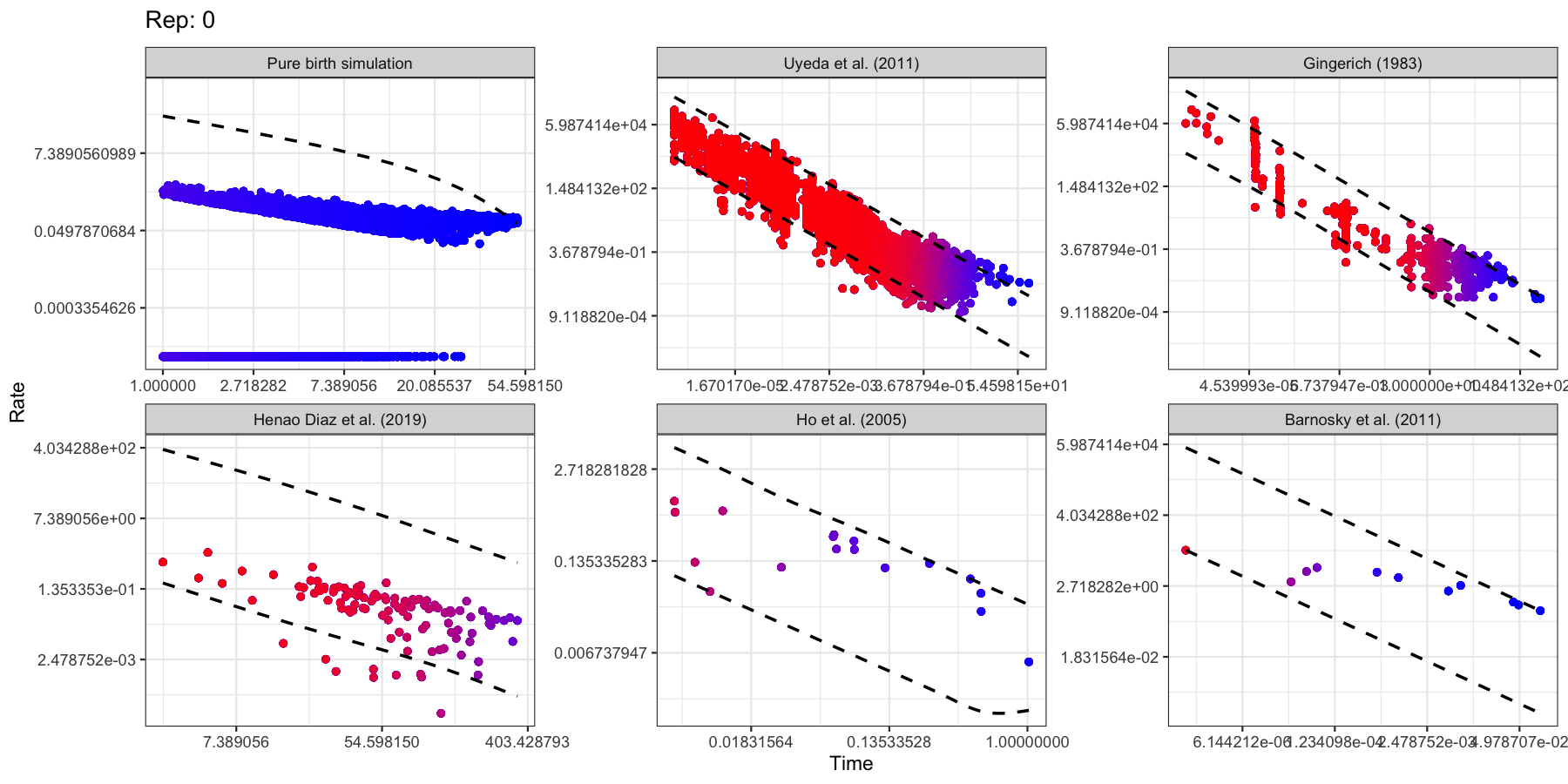

Supplement: S1 Movie — (GIF) [file pcbi.1012458.s015.gif]
